# Podocyte Autophagy: A Potential Therapeutic Target to Prevent the Progression of Diabetic Nephropathy

**DOI:** 10.1155/2017/3560238

**Published:** 2017-04-23

**Authors:** Na Liu, Liuqing Xu, Yingfeng Shi, Shougang Zhuang

**Affiliations:** ^1^Department of Nephrology, Shanghai East Hospital, Tongji University School of Medicine, Shanghai, China; ^2^Department of Medicine, Rhode Island Hospital and Alpert Medical School, Brown University, Providence, RI, USA

## Abstract

Diabetic nephropathy (DN), a leading cause of end-stage renal disease (ESRD), becomes a worldwide problem. Ultrastructural changes of the glomerular filtration barrier, especially the pathological changes of podocytes, lead to proteinuria in patients with diabetes. Podocytes are major components of glomerular filtration barrier, lining outside of the glomerular basement membrane (GBM) to maintain the permeability of the GBM. Autophagy is a high conserved cellular process in lysosomes including impaired protein, cell organelles, and other contents in the cytoplasm. Recent studies suggest that activation of autophagy in podocytes may be a potential therapy to prevent the progression of DN. Here, we review the mechanisms of autophagy in podocytes and discuss the current studies about alleviating proteinuria via activating podocyte autophagy.

## 1. Introduction

Diabetes mellitus (DM) has been one of the global health issues. According to the report from the International Diabetes Federation, the number of patients with DM will increase to 205 million in 2035 than in 2014. Diabetic nephropathy (DN), a serious chronic complication of DM, is a leading cause of end-stage renal disease (ESRD). One significant clinical feature of DN is the appearance of urinary protein, defined as “albuminuria.” Structural changes of the glomerular filtration barrier are detected in the diabetic patients with albuminuria, including glomerular endothelial cell injury, the loss of podocytes, glomerular basement membrane (GBM) thickening, and mesangial expansion [1, 2]. Apart from GBM dysfunction, the accumulation of advanced glycation end products (AGEs), oxidative stress, and the activation of the renin-angiotensin system (RAS) also contribute to the decline in renal function [1, 3–7].

Based on the pathologic alterations in the kidney, DN is classified into four groups: class I includes GBM thickening, class II consists of mild (IIA) to severe (IIB) mesangial expansion, class III includes nodular glomerulosclerosis, and class IV represents developed DN which is characterized with over 50% global glomerulosclerosis and podocyte deficiency [8, 9]. Among these four categories, the kidney may also exhibit arteriolar hyalinosis, glomerular capillary subendothelial hyaline, and arteriosclerosis. Present therapies are mainly focusing on the way to reduce the levels of blood glucose and blood pressure to normal, and most of them alleviate albuminuria via suppressing the RAS activity [10]. Nevertheless, considering the elevation of diabetic kidney diseases, further studies in a pathogenetic mechanism for DN are needed to find new approaches to treat DN. Recently, a number of reports have demonstrated that autophagy is involved in the pathogenesis of diabetes-related podocyte injury. In this review, we will make a summary on the role of autophagy in this process and the mechanisms involved.

## 2. Autophagy

Autophagy (from the ancient Greek meaning “self-eating”) is a highly conserved cellular process that delivers protein and other impaired cell organelles to lysosomes for degradation and recycle to maintain intracellular homeostasis. Christian de Duve first referred autophagy in 1963 [11]. Subsequent studies focused on the regulatory mechanisms of autophagy and its effects on human health and disease.

On the basis of different ways of transporting intracellular constituents to lysosomes, autophagy is divided into three types: macroautophagy, microautophagy, and chaperone-mediated autophagy [12]. Macroautophagy and chaperone-mediated autophagy are through autophagosomes and chaperone complex, respectively, while constituents are delivered to lysosomes directly in microautophagy [13, 14]. In this review, macroautophagy (hereafter referred to as autophagy) is mainly investigated among these three types. In terms of different types of degraded substrates, autophagy was also divided into selective and nonselective autophagy. Degradation of some impaired organelles, lipophagy, or xenophagy is involved in selective autophagy, whereas deficient nutrient-induced autophagy is considered the nonselective type [15–17].

Autophagy, first detected in the yeast, is a complex process comprising of autophagy-related gene (Atg) product cooperation. Atg proteins are classified into five groups: Atg1 kinase complex [Atg1/Unc-51-like kinase (ULK) 1/2], Atg9, class III phosphoinositide 3-kinase complex (PI3KC3), and two ubiquitin-like conjugation systems (Atg12-Atg5 and Atg8 conjugation system) [18]. Besides the Atg regulation, there are some other regulatory mechanisms of autophagy, such as the mammalian target of rapamycin signaling pathway and cellular stress pathway [19–21].

## 3. Podocyte Autophagy in Diabetic Nephropathy

Studies have demonstrated that autophagy is renoprotective in acute kidney injury, obstructive nephropathy, diabetic nephropathy, and other renal diseases [22]. Podocytes are highly differentiated epithelial cells lining the outer aspect of the GBM with interdigitating foot processes, and the slit diaphragms between foot processes play a role in substance filtration. Podocyte injury including foot process fusion and slit diaphragm alteration results in abnormal permeability of the GBM, terminally leading to albuminuria. Autophagy controls the quality of the cytoplasm, via degrading proteins, peroxidases, and damaged organelles that complicate the recycle of organelles, and then maintains the homeostasis of intracellular environment [23, 24]. The self-repaired feature of autophagy is important in the anaphase cells such as neurocytes and podocytes, which have a restricted capacity in differentiation and proliferation [25]. The previous studies explored the mechanisms of podocyte autophagy in DN and suggested that activated podocyte autophagy has an effect on DN through an Atg12-Atg5 conjugation system, mTOR, adenosine 5′-monophosphate- (AMP-) activated protein kinase (AMPK), and oxidative stress as well as vascular endothelial growth factor.

### 3.1. Atg12-Atg5 Conjugation System in Podocyte Autophagy and Diabetic Nephropathy

Atg12 is a ubiquitin-like protein involving in autophagosome formation. Autophagy activation needs the conjugation of Atg12 to Atg5, which is stimulated by Atg7 and Atg10, and then promotes Atg8 and lipid phosphatidylethanolamine conjugation in the cytoplasm [26]. The activation of the Atg12-Atg5 conjugation system promotes the production of autophagosome and then activates podocyte autophagy. Currently, Liu et al. demonstrated that *β*-arrestins, a negative adaptor of G protein-coupled receptors (GPCRs), aggravate podocyte injury through autophagy inhibition in DN [27]. They found that *β*-arrestins suppressed podocyte autophagy via downregulating Atg12-Atg5 conjugation, which is induced by enhancing the interaction between *β*-arrestins and Atg7. Therefore, modulation of this pathway may be a novel therapeutic approach for treating patients with DN.

### 3.2. mTOR Signaling Pathway in Podocyte Autophagy and Diabetic Nephropathy

Mammalian target of rapamycin (mTOR) is essential to cell growth regulation, and activation of mTOR suppresses autophagy. Deficient nutrients (such as growth factor or amino acid deficiency) in the cytoplasm activate autophagy by suppressing the expression of mTOR. After inhibition, mTOR not only can activate the formation of class III phosphatidylinositol 3-kinase (PI3K) complex and the unc-51-like kinase (Ulk) 1 complex but also inhibit the activity of ribosome protein subunit 6 kinase 1 (S6K1) [28–31]. In the upstream of mTOR, there are two separated protein kinases, phosphatidylinositol 3-kinase I (PI3K-I)/protein kinase B and AMP-activated protein kinase, which are regulated by different conditions [32].

#### 3.2.1. Phosphatidylinositol 3-Kinase I (PI3K-I)/Protein Kinase B (Akt/PKB)

PI3Ks are consisted of three isoforms, including class I, class II, and class III [33, 34]. As a member of Atg proteins, class III PI3K composes of a Vps15 regulatory subunit and a Vps34 catalytic subunit, which promote phosphatidylinositol (PI) conversion to phosphatidylinositol 3-phosphate [PI(3)P] and then initiate autophagy [35–38]. In contrast, the class I PI3K regulatory subunit p58 is bonded to the catalytic subunit p110 and then activates the Akt/mTOR signaling pathway [39, 40] by promoting phosphatidylinositol 3,4,5-triphosphate. Therefore, it seems that class I PI3K inhibits autophagy while class III PI3K activates it. The activation of class I PI3K is triggered by insulin or growth factors to interact with insulin receptors or tyrosine kinase receptors, which are the members of transmembrane receptors existing on the membrane of podocytes and then activates Akt/PKB. Then, the downstream tuberous sclerosis complex 1 and 2 (TSC1/2) proteins will be inhibited by PKD1 and the production of Akt/PKB activation. In the end, podocyte autophagy is suppressed by the activation of mTOR.

Recent studies have emphasized the relationship between DN and nutrient-dependent pathways, involving the mTOR signaling pathway. In the models of diabetic nephropathy, especially the type 1, insulin resistance blocks the phosphorylation of Akt/PKB and then activates mTOR by increasing the expression of Rheb (Ras homolog enriched in brain). Thus, insulin resistance suppresses podocyte autophagy through increasing the activity of mTOR.

#### 3.2.2. AMP-Activated Protein Kinase (AMPK)

**Figure 1:**
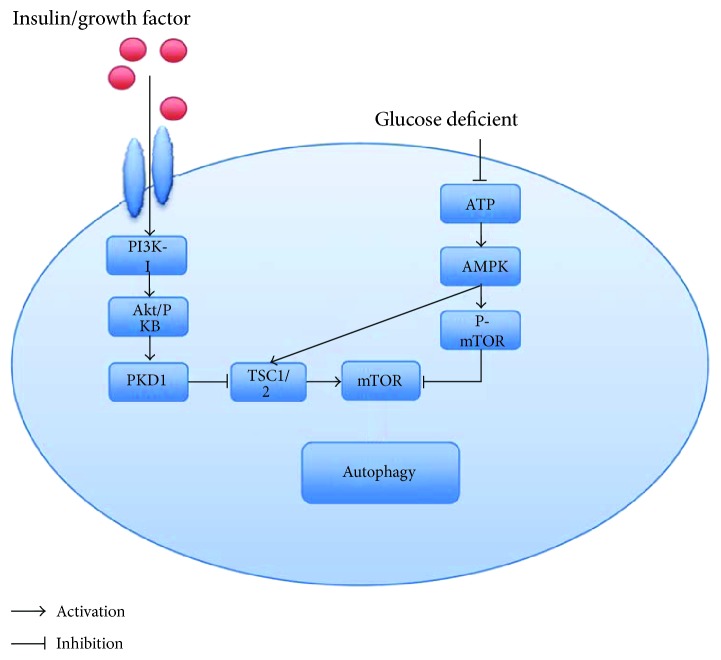
mTOR signaling pathway in podocyte autophagy. PI3K-I: class I phosphatidylinositol 3-kinase; Akt/PKB: protein kinase B; TSC: tuberous sclerosis complex; ATP: adenosine triphosphate; AMPK: AMP-activated protein kinase.

As an essential regulator in energy metabolism, AMPK is an enzyme consisted of three proteins (*α*, *β*, and *γ*) [41]. AMPK can be activated by an increase in Ca^2+^ concentration in the cytoplasm [42, 43] and the stimulation of numerous hormones, adipokines, and cytokines. In addition to these, the ratio of an intracellular AMP/ATP decrease also activates AMPK. Nutrient starvation induced the activation of AMPK. In the condition of ATP deficiency, the downstream TSC1/2 is activated by AMPK, then inhibits Rheb, and finally enhances autophagy by suppressing mTOR activation (Figure 1). Recently, Jin et al. suggested that berberine alleviated high glucose-induced apoptosis of podocytes in mouse via increasing the activity of AMPK [44]. They showed that the expression of p-AMPK in a high-glucose (HG) group was lower and the expression of p-mTOR was higher in the HG group compared with the control group, while these results were conversed by berberine administration.

Mechanical stress induced by the renin-angiotensin system is considered a major damage factor in podocytes of DN. Spironolactone, a common diuretic, is generally used to treat heart failure, edema, or Conn's syndrome. The study from Li et al. demonstrated that spironolactone has renoprotective effects on activating autophagy through blockage of the mTOR signalling pathway in podocytes under mechanical stress [45]. They used the Flexercell FX-5000TM Compression System to establish the animal model of DN and found that the expressions of p85-PI3K, p-AKT, and p-mTOR were significantly increased compared with those of the control group. After administration of spironolactone for 48 h, the levels of p85-PI3K, p-AKT, and p-mTOR were markedly decreased, which are in accordance with the results in the group by PI3K inhibition. Thus, spironolactone might be a new therapy of DN.

Rapamycin is a new immunosuppressive drug of macrocyclic lactone, which was first found in a soil bacterium in 1965 [46]. After that, researchers suggested that rapamycin has antifungal effects as well as anti-T cell activity in succession [47]. Furthermore, rapamycin is a selective inhibitor of mTOR [48]. Rapamycin binding to immunophilins, such as FKBP12 (*FK* binding protein, 12 kDa), forms an FKBP12-rapamycin complex. The FKBP12-rapamycin complex suppresses the expression of mTOR through phosphorylation of mTOR and then activates autophagy. However, the number of clinical trials of rapamycin in DN is less; further studies are needed to clarify the renoprotective property of rapamycin in DN.

### 3.3. Reactive Oxygen Species (ROS) in Podocyte Autophagy and Diabetic Nephropathy

**Figure 2:**
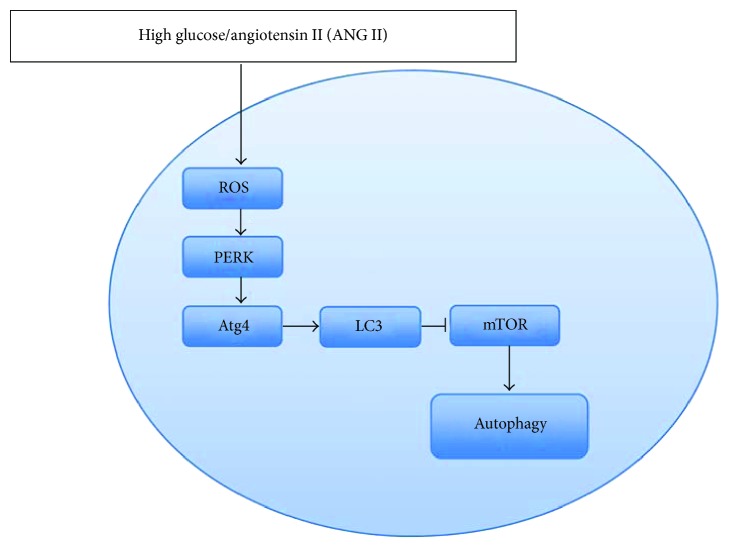
Reactive oxygen species (ROS) in podocyte autophagy. ROS: reactive oxygen species; PERK: PKR-like endoplasmic reticulum kinase.

Besides insulin and nutrition starvation, intracellular metabolism alternations are also related to the pathogenesis of DN, involving the increase in reactive oxygen species (ROS). Several studies have shown that ROS are the most common factors in activating podocyte autophagy. An increase in ROS production activates PKR-like kinase (PERK), which then oxidizes Atg4 proteases via eIF2a phosphorylation, subsequently promotes the level of proteolytic mature LC3, and prevents mTOR activation [49] (Figure 2). Recently, Ma et al. explored the effect of high-glucose milieu on podocyte autophagy and suggested that podocyte autophagy was activated by upregulating the generation of mitochondrial ROS after exposing to high glucose for 24 hours [50]. Meanwhile, podocytes exposed to angiotensin II (ANGII) also increased the generation of ROS and promoted autophagy activation [51]. However, the membrane of the mitochondrion is damaged by excessive ROS generation in the mitochondrion, and ROS releasing into the cytoplasm may cause damage to other organelles. Since the function of autophagy targeting and degrading injury organelles is selective, the augmentation of ROS will be limited [52]. Chronic exposure to high-glucose condition leads to autophagy insufficiency and subsequently causes lysosomal dysfunction and podocyte apoptosis, finally resulting in diabetic nephropathy [53]. Therefore, reduction of ROS generation is a potential therapeutic approach for preventing the development of DN.

### 3.4. Vascular Endothelial Growth Factor (VEGF) in Podocyte Autophagy and Diabetic Nephropathy

In the early phases of animals or patients with DN, the level of VEGF has been shown to be increased in the kidney. Several studies have suggested that elevation of VEGF is associated with the increase in the glomerular permeability, then resulting in proteinuria [54].VEGF is considered to be a promoter of angiogenesis and synthesized mainly by the podocytes. VEGF-A, as one member of a VEGF family, has a negative effect on glomerular endothelial cell (GEC) glycocalyx through the early stages of DN, and this effect can be reversed by VEGF-A_165b_, an inhibitory isoform of VEGF-A, finally ameliorating proteinuria [55]. Autophagy has been reported to prevent angiogenesis [56, 57]. Miaomiao et al. found that high glucose enhanced the level of VEGF, whereas this elevation is downregulated by autophagy activation via rapamycin, an inhibitor of mTOR [58]. Yang [59] and Liu et al. [60] also demonstrated that the increase in autophagosome inhibits angiogenesis.

## 4. Conclusion

According to the International Diabetes Federation, the global diabetes prevalence will increase from 8.3% in 2014 to 10.1% in 2053. As a serious global health issue, it is urgent to find potent therapies to treat diabetes and its complications, especially diabetic nephropathy. The previous studies have shown the activation of autophagy in podocytes via inhibiting the expression of mTOR and alleviating albuminuria in DN. Meanwhile, autophagy activation also decreased the expression of VEGF and subsequently prevented the progression of DN. Although studies have suggested that podocyte autophagy is a renoprotective process in lysosome, DN is an extremely complex complication. Further investigations are needed to elucidate the role of autophagy in podocyte injury induced by DN and discover the autophagy-based therapies for the treatment of DN.
